# 2.5D peritumoural radiomics predicts postoperative recurrence in stage I lung adenocarcinoma

**DOI:** 10.3389/fonc.2024.1382815

**Published:** 2024-08-29

**Authors:** Haimei Lan, Chaosheng Wei, Fengming Xu, Eqing Yang, Dayu Lu, Qing Feng, Tao Li

**Affiliations:** ^1^ Department of Radiology, Liuzhou Workers Hospital, Liuzhou, Guangxi, China; ^2^ Department of Radiology, Longtan Hospital, Liuzhou, Guangxi, China

**Keywords:** radiomics, lung adenocarcinoma, postoperative recurrence, nomogram, peritumoral regions

## Abstract

**Objective:**

Radiomics can non-invasively predict the prognosis of a tumour by applying advanced imaging feature algorithms.The aim of this study was to predict the chance of postoperative recurrence by modelling tumour radiomics and peritumour radiomics and clinical features in patients with stage I lung adenocarcinoma (LUAD).

**Materials and methods:**

Retrospective analysis of 190 patients with postoperative pathologically confirmed stage I LUAD from centre 1, who were divided into training cohort and internal validation cohort, with centre 2 added as external validation cohort. To develop a combined radiation-clinical omics model nomogram incorporating clinical features based on images from low-dose lung cancer screening CT plain for predicting postoperative recurrence and to evaluate the performance of the nomogram in the training cohort, internal validation cohort and external validation cohort.

**Results:**

A total of 190 patients were included in the model in centre 1 and randomised into a training cohort of 133 and an internal validation cohort of 57 in a ratio of 7:3, and 39 were included in centre 2 as an external validation cohort. In the training cohort (AUC=0.865, 95% CI 0.824-0.906), internal validation cohort (AUC=0.902, 95% CI 0.851-0.953) and external validation cohort (AUC=0.830,95% CI 0.751-0.908), the combined radiation-clinical omics model had a good predictive ability. The combined model performed significantly better than the conventional single-modality models (clinical model, radiomic model), and the calibration curve and decision curve analysis (DCA) showed high accuracy and clinical utility of the nomogram.

**Conclusion:**

The combined preoperative radiation-clinical omics model provides good predictive value for postoperative recurrence in stage ILUAD and combines the model’s superiority in both internal and external validation cohorts, demonstrating its potential to aid in postoperative treatment strategies.

## 1 Introduction

Lung cancer is a significant contributor to global cancer mortality (1). Lung cancer is classified into two main groups (2, 3): non-small cell lung cancer (NSCLC) and small cell lung cancer (SCLC), of which about 85% of patients belong to NSCLC, which includes lung adenocarcinoma (LUAD), lung squamous carcinoma (LUSC), and other histological subtypes. In NSCLC patients, LUAD accounts for the largest proportion. With the development of low-dose computed tomography(LDCT) lung cancer screening, a large number of patients with early-stage NSCLC have been screened, and in particular, a considerable number of patients with stage I LUAD have been screened (4, 5), for which surgical resection is the preferred treatment (6). However, studies have found that the risk of recurrence remains high, even with a 20-50% recurrence rate for completely resected stage I LUAD (7). Therefore, assessment of postoperative recurrence is crucial for the prognosis of stage I LUAD.

Currently, most studies have focused on assessing benign and malignant tumours (8), disregarding the prognostic impact of subtle changes in the peritumoural microenvironment (9, 10). Furthermore, studies on the prognosis of LUAD have primarily concentrated on evaluating the prognosis of intermediate and advanced lung cancer based on genes and treatment regimens (11–14), while neglecting the impact of certain clinical factors such as immunohistochemistry and density on the prognosis. It is important to note that due to the heterogeneity of tumours (15, 16), even at the same stage, the prognosis can vary significantly. Moreover, most of the previous studies have been on two-dimension (2D) and three-dimension (3D) prognostic models (17, 18), and nowadays some scholars have started to study 2.5-dimension (2.5D) models (19) as well. Through the peritumoural radiomics prognostic study of stage I LUAD (20, 21), this study not only makes up for the shortcomings of previous studies, but also develops a new 2.5D peritumoural radiation-clinical omics model. Compared with previous 2D or 3D radiomics features, the method is newer and more effective in studying the prognosis of LUAD.

## 2 Materials and methods

### 2.1 Patient selection and follow-up

This retrospective study was approved by two institutional review boards of the Guangxi Zhuang Autonomous Region (NO.LW2024009), exempting patients from informed consent. We collected medical records of all patients with stage I LUAD who underwent surgical resection and were pathologically confirmed between January 2010 and December 2018 at the centre 1. The inclusion criteria (1): underwent surgical complete resection of the lung lesion (2); postoperative pathological diagnosis of invasive stage I lung adenocarcinoma (3); CT examination within 2 weeks before surgery. The exclusion criteria (1): the presence of multiple primary cancers or other malignancies in the lungs (2); preoperative neoadjuvant therapy (3); failure to complete postoperative follow-up (4); CT image artefacts that severely impaired the visualisation of the tumour (5); absence of low-dose lung cancer screening CT plain images prior to surgery.

A total of 190 patients with stage I LUAD were included in centre 1 and randomised into two cohorts in a ratio of 7:3. The training cohort consisted of 133 patients, while the internal validation cohort had 57 patients. Additionally, 39 patients with stage I LUAD in centre 2 were collected as the external testing cohort from January 2016 to December 2018, following the same inclusion and exclusion criteria. A postoperative follow-up was conducted, including computed tomography (CT) and/or magnetic resonance imaging (MRI), PET-CT. Recurrence was defined as local recurrence and distant metastasis, as per relevant studies. Local recurrence included recurrence in N1 lymph nodes, N2 lymph nodes, mediastinum, primary lung or pleura. Distant metastases included metastases to the adrenal gland, kidney, bone, brain, liver, contralateral lung, skin or N3 (22).

### 2.2 Clinical characteristic

Basic patient information and clinical variables including age, sex, white blood cell (WBC), neutrophils (NEU), C-reactive protein (C-RP), carcinoembryonic antigen (CEA), cytokeratin 19 fragment assay (CYFRA21-1), neuron-specific enolase assay (NSE), carbohydrate antigen (CA) 125, CA153, squamous cell carcinoma-associated antigen (SCCA), CA50, CA242, CA724, Ki-67, location of the tumour, distance from the pleura, T-stage, and type of nodule.

We divided the age into two groups: less than 65 years old and greater than or equal to 65 years old; T-stage was determined by experienced radiologists from preoperative CT images, based on the 9th edition of the TNM staging system for lung cancer, and was divided into T1a, T1b, and T1c; the division of the content of Ki67 is still controversial, and we used less than 10% for low expression and greater than or equal to 10% for high expression; and the type of nodules of stage I LUAD that we included showed mixed ground glass nodules (mGGN) and solid nodules (SN).

### 2.3 Procedure

**Figure 1:**
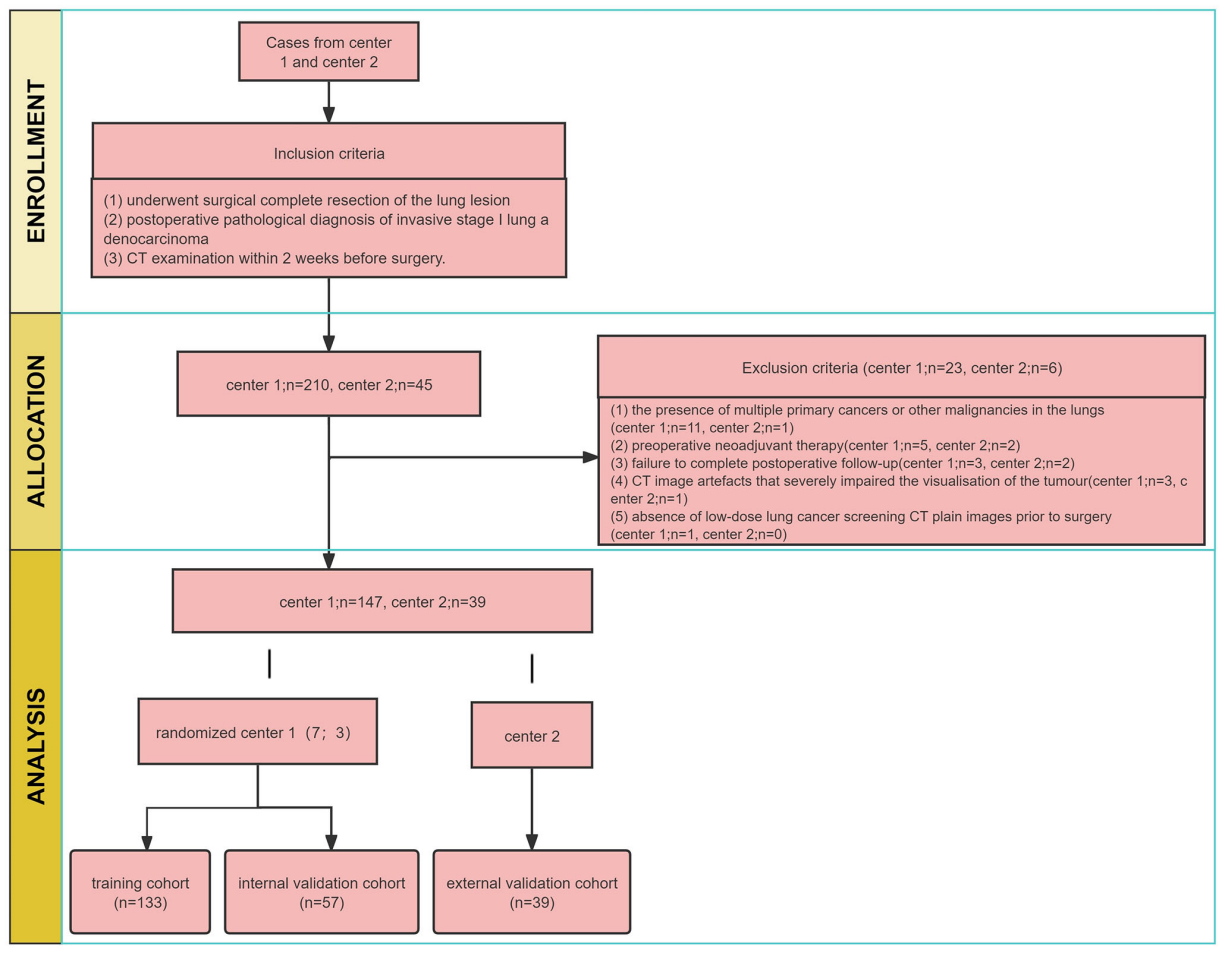
Flow diagram of the study population.

**Figure 2:**
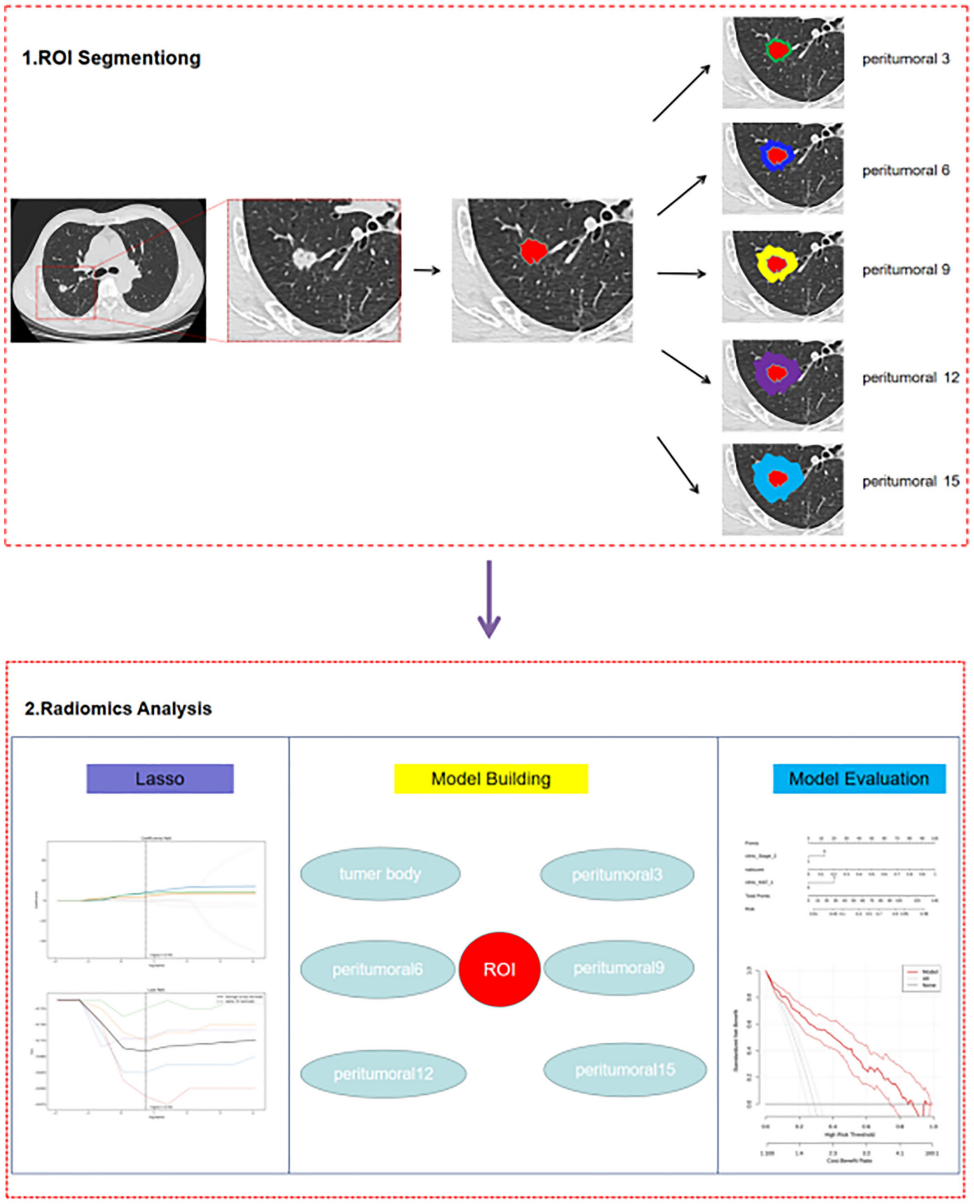
The radiomics modelling pipeline.

The study workflow is summarized in **Figure 1**, and the radiomics modelling pipeline in **Figure 2**.

### 2.4 CT image acquisition

The scanning machine at both hospitals was SIEMENS SOMATOM Definition Flash (Stellar) with the same lung scanning parameters. All CT scans were performed from the tip of the lungs to the base of the lungs, and the parameters of the scan reconstruction were: Tube voltage=120kV, Effective power of tube=30mAs, Detector collimation=128 × 0.625mm, Matrix=512×512, Slice thickness=0.625mm, CDTIvol=2.03mGy.

### 2.5 Radiomics feature extraction and feature selection

**Figure 3:**
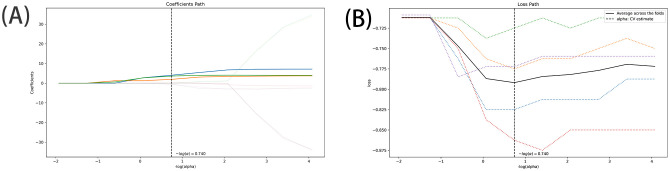
Feature selection using the LASSO algorithm [**(A)**, LASSO path; **(B)**, MSE path].

**Figure 4:**
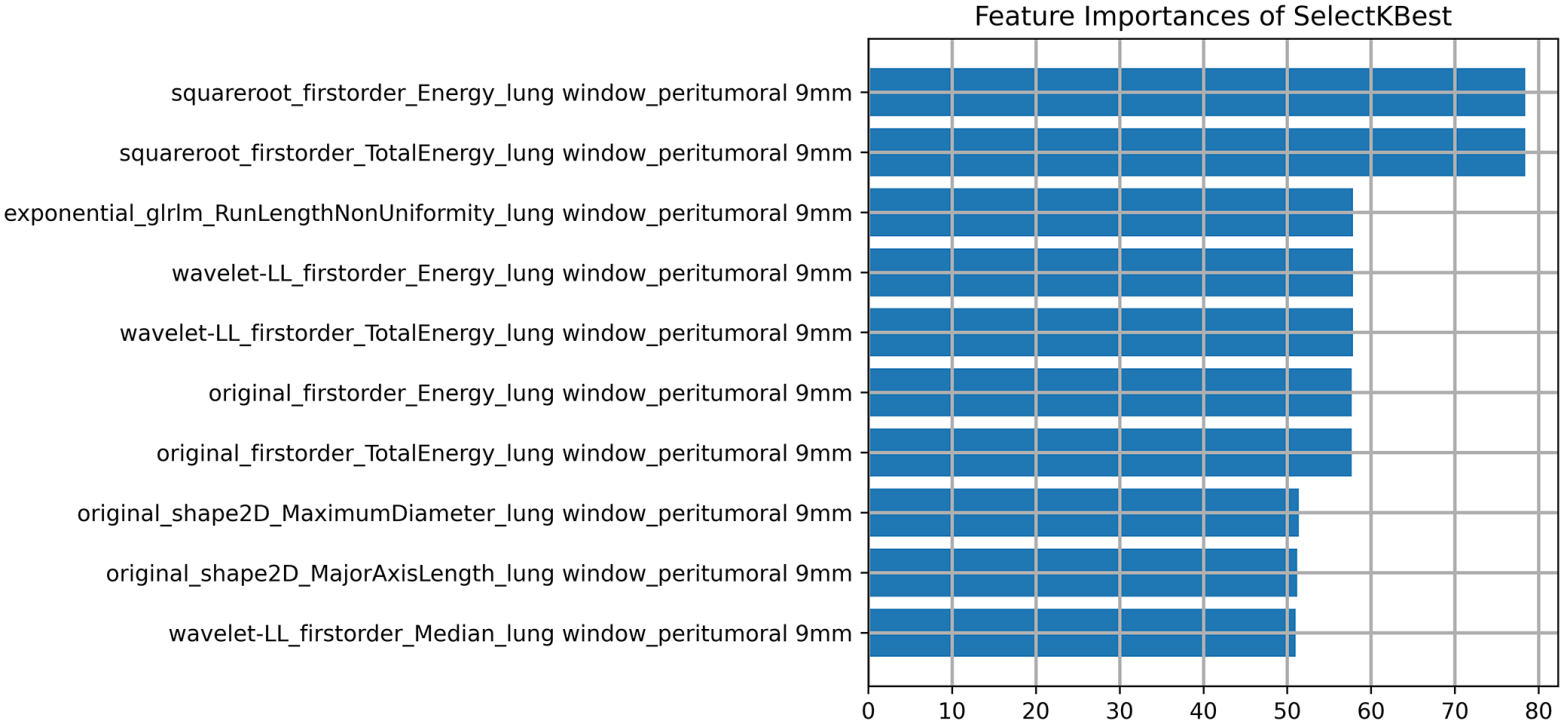
The final 10 features selected (10 textures).

The DICOM format images of the patients were downloaded from the Picture Archiving and Communication Systems (PACS) and imported into the Darwin Intelligent Science Research Platform. The process of tumour region segmentation and radiomics feature extraction involves the following steps (1): Modal settings: the modal parameters for each patient were set to tumour body, peritumoural 3mm, peritumoural 6mm, peritumoural 9mm, peritumoural 12mm and peritumoural 15mm, and the window widths and window positions were uniformly set to 1200 and -600 (2); 2.5D region of interest (ROI) segmentation: The ROI was manually delineated on the CT images by two radiologists with 10 years of experience. For each CT image, the radiologist selected the largest section of the tumour on the Darwin Intelligent Science research platform (23) to draw a ROI, and then selected the forward and backward angles of 45° on this section to draw two ROIs. These three ROIs were then merged to create a 2.5D ROI for each tumour. In outlining ROIs, we exclude pleural walls, thick bronchial tubes, and blood vessels (3); A total of 1125 radiomics features were extracted using the Darwin Intelligent Science Research Platform (4); A minimum-maximum normalised, optimal feature filter was used to assess the linear correlation between each feature and the lesion category labels, and the 40 most relevant features were filtered out of 1125 features. The least absolute shrinkage and selection operator (LASSO) algorithm was used to select the most relevant features from 40 features (**Figure 3**). Finally, a total of 10 features most relevant to recurrence after surgery for stage I LUAD were selected and used to construct a prediction model (**Figure 4**).

### 2.6 Intra-observer and inter-observer consistency

We used intraclass correlation coefficient (ICC) to assess intra- and inter-observer correlation coefficients. A total of 49 patients were randomly selected from the training set, and ROI segmentation was independently performed by two physicians. We considered these features to be stable when the ICC value was greater than 0.80.

### 2.7 Model construction and validation

To predict postoperative recurrence of stage I LUAD, we performed univariate and multifactorial logistic regression (LR) analyses to select statistically significant clinical characteristics (P < 0.05) for clinical modelling. Clinical characteristics that were statistically significant for postoperative recurrence of stage I LUAD were retained in the univariate logistic regression analysis (p<0.05). Variables with p<0.05 in the multifactorial analysis were identified as independent predictors associated with postoperative recurrence and were included in the construction of the clinical model. Combining the radiomics model with the clinical model to create a joint model with different parameters. Receiver operating characteristic (ROC) curves were plotted, and area under the receiver operating characteristic curve (AUC) was calculated to assess the efficacy of each model. We compared thirteen predictive models, including six parameter radiomics models, clinical model, and six radiation-clinical omics models incorporating clinical factors. The best models were then selected from these to draw nomogram, and we used deLong tests, calibration curves, and decision curve analyses (DCA) to test the accuracy and clinical utility of the nomogram.

### 2.8 Statistical analysis

SPSS 24.0 was used for statistical analysis. Continuous variables were presented as mean ± standard deviation and compared using independent samples t-test. Categorical variables were presented as percentage counts and compared using chi-square test. The model’s goodness of fit was assessed using the Hosmer-Lemeshow test, which showed no statistically significant difference (P > 0.05), indicating good model fit. To comprehensively evaluate the predictive efficacy of different models, we used ROC curve, AUC, accuracy, sensitivity, specificity, Positive predictive value(PPV), and Negative predictive value (NPV). All statistical tests were two-sided with a significance level of p<0.05.

## 3 Results

### 3.1 Patient clinical baseline characteristics

**Table 1** lists and compares the clinical baseline characteristics of the analysed patients.

**Table 1:** Clinical baseline characteristics.

	Training Set (n1 = 133)	Internal Test Set (n2 = 57)	p value (n1 VS n2)	External Test Set (n3 = 39)	p value (n1 VS n3)
Age			0.838		0.571
<65	88(66.2)	43 (75.4)		25 (64.1)	
≥65	45(33.8)	14 (24.6)		14 (35.9)	
Sex			0.777		0.705
Male	66 (49.6)	27 (47.4)		18 (46.2)	
Female	67 (50.4)	30 (52.6)		21 (53.8)	
Stage			0.213		0.371
T1a	8 (6.0)	7 (12.2)		4 (10.2)	
T1b	77 (57.9)	38 (66.7)		23 (59.0)	
T1c	48 (36.1)	12 (21.1)		12 (30.8)	
WBC (Mean ± SD)	6.97 ± 2.34	6.93 ± 2.06	0.911	6.77 ± 1.87	0.707
NEU (Mean ± SD)	4.30 ± 2.04	4.14 ± 1.48	0.604	3.99 ± 1.53	0.441
C-RP (Mean ± SD)	6.08 ± 14.58	2.66 ± 3.63	0.093	4.56 ± 14.98	0.585
CEA (Mean ± SD)	5.38 ± 7.90	3.82 ± 4.14	0.082	7.02 ± 18.99	0.431
CYFRA21-1 (Mean ± SD)	3.09 ± 1.74	3.55 ± 2.28	0.175	2.97 ± 1.86	0.776
NSE (Mean ± SD)	13.26 ± 3.69	13.93 ± 6.19	0.392	13.18 ± 4.74	0.792
CA125 (Mean ± SD)	15.29 ± 12.00	12.03 ± 16.85	0.156	14.09 ± 15.03	0.678
CA15-3 (Mean ± SD)	15.68 ± 15.77	13.17 ± 9.26	0.294	19.78 ± 16.23	0.318
SCCA (Mean ± SD)	1.19 ± 0.79	1.42 ± 0.86	0.088	1.55 ± 0.79	0.335
CA50 (Mean ± SD)	9.56 ± 14.43	23.93 ± 71.84	0.163	8.32 ± 9.80	0.568
CA242 (Mean ± SD)	6.59 ± 6.10	12.56 ± 34.26	0.222	5.09 ± 4.91	0.205
CA72-4 (Mean ± SD)	4.42 ± 9.16	3.82 ± 7.78	0.682	5.04 ± 13.42	0.750
Ki67			0.078		0.095
<10%	68 (51.1)	26 (45.6)		14 (35.9)	
≥10%	65 (48.9)	31 (54.4)		25 (64.1)	
Location			0.854		0.580
Left superior lobar	37 (27.8)	15 (26.3)		14 (35.9)	
Right superior lobar	45 (33.8)	20 (35.1)		10 (25.7)	
Right middle lobar	8 (6.0)	4 (7.0)		7 (17.9)	
Right inferior lobar	27 (20.3)	14 (24.6)		2 (5.1)	
Left inferior lobar	16 (12.0)	4 (7.0)		6 (15.4)	
Distance from pleura (Mean ± SD)	1.45 ± 0.73	1.50 ± 0.65	0.640	1.50 ± 0.65	0.402
Nodule type			0.920		0.079
mGGN	43 (32.3)	18 (31.6)		18 (9.5)	
SN	90 (67.7)	39 (68.4)		39 (20.5)	
Recurrence			0.862		0.628
Yes	39 (29.3)	16 (28.1)		11 (28.2)	
No	94 (70.7)	41 (71.9)		28 (71.8)	

### 3.2 Establishment of clinical models

Logistic regression analysis was used to assess 19 possible risk factors. Univariate and multifactorial logistic regression analyses were performed on clinical indicators in training cohort of 133 patients with postoperative recurrence of stage I LUAD (**Table 2**). Univariate logistic regression analysis showed that T1c in T-stage, CEA, NSE, ≥10% in Ki67, and SN in nodal type were statistically significant for postoperative recurrence of stage ILUAD. For statistically significant clinical characteristics, multifactorial logistic regression analysis was used, which showed that NSE, ≥10% in Ki67, T-stage in T1c and SN in nodule type were independent risk factors for postoperative recurrence and could be used to establish clinical models.

**Table 2:** Univariate and multivariate analysis.

	N(100%)	OR(95%CI)	p value	OR(95%CI)	p value
Age					
<65	88(66.2)	1.000			
≥65	45(33.8)	0.969(0.439-2.136)	0.937		
Sex					
Male	66(49.6)	1.000			
Female	67(50.4)	1.270(0.601-2.685)	0.531		
Stage					
T1a	8(6.0)	1.000			
T1b	77(7.9)	3.500(0.437-28.004)	0.238	4.092(0.497-33.707)	0.190
T1c	48(36.1)	4.549(2.135-9.695)	**0.000**	14.237(1.704-118.970)	**0.014**
WBC		1.123(0.962-1.311)	0.141	–	–
NEU		1.165(0.974-1.392)	0.095	–	–
C-RP		1.013(0.989-1.039)	0.293	–	–
CEA		1.095(1.020-1.175)	**0.012**	–	–
CYFRA21-1		1.182(0.943-1.481)	0.147	–	–
NSE		1.126(1.005-1.263)	**0.041**	1.215(1.032-1.430)	**0.020**
CA125		1.020(0.988-1.052)	0.223	–	–
CA153		1.013(0.989-1.038)	0.304	–	–
SCCA		0.752(0.428-1.320)	0.320	–	–
CA50		1.010(0.985-1.036)	0.430	–	–
CA242		1.054(0.991-1.122)	0.097	–	–
CA724		1.028(0.984-1.073)	0.216	–	–
Ki67					
<10%	68(51.1)	1.000			
≥10%	65(48.9)	10.656(4.044-28.078)	**0.000**	0.081(0.020-0.322)	**0.000**
Location					
Left superior lobar	37(27.8)	1.000		–	–
Right superior lobar	45(33.8)	0.758(0.292-1.967)	0.568	–	–
Right middle lobar	8(6.0)	1.250(0.255-6.119)	0.783	–	–
Right inferior lobar	27(20.3)	0.595(0.191-1.859)	0.372	–	–
Left inferior lobar	16(12.0)	1.250(0.368-4.251)	0.721	–	–
Distance from pleura		0.822(0.470-1.439)	0.492	–	–
Nodule type					
mGGN	43(32.3)	1.000			
SN	90(67.7)	6.205(2.039-18.881)	**0.001**	4.541(1.716-12.014)	**0.002**

mGGN, Mmixed ground-glass nodule; SN, Solid nodules; SD, Standard deviation. Bolded indicators are meaningful. Values in bold indicate statistical significance.

### 3.3 Performance and comparison of models

**Figure 5:**
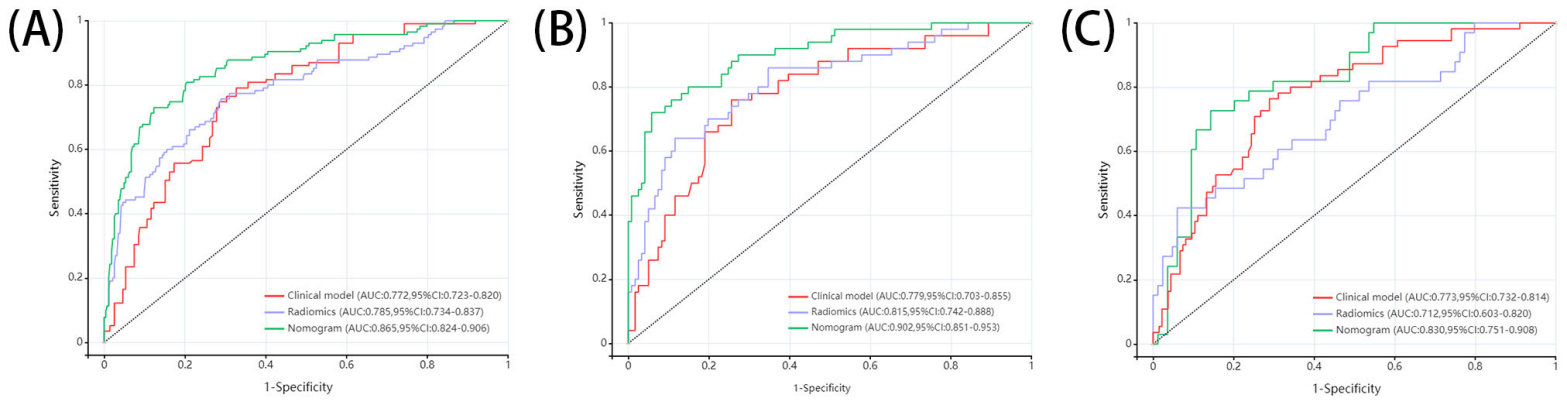
ROC curves of peritumoral 9mm model, clinical model, and combined radiation-clinical omics model in training cohort **(A)**, internal validation cohort **(B)**, and external validation cohort **(C)**.

**Figure 6:**
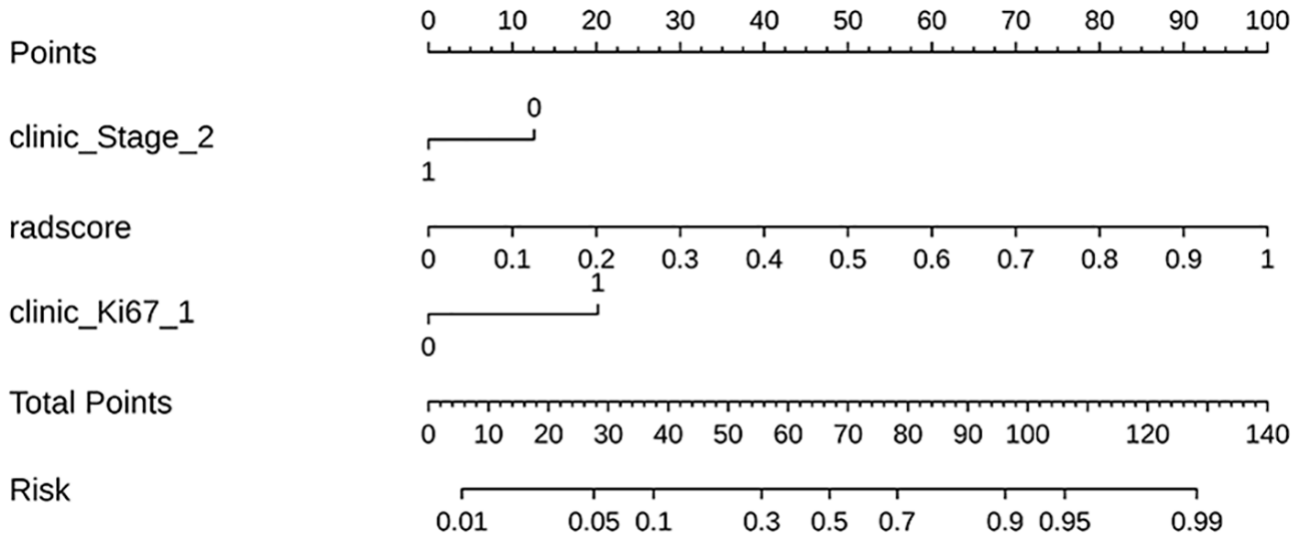
The radiomics nomogram incorporating some of the independent risk factors; the peritumoral 9mm model’s radscore = +4.611 *wavelet-LL_first order_Median_lung window_peritumoral 9mm + 4.114 *original_shape 2.5D_MaximumDiameter_lung window_peritumoral 9mm-4.119.

In this study, we developed 13 models, including the radiomics models with 6 parameters (tumour body, peritumoural 3mm, peritumoural 6mm, peritumoural 9mm, peritumoural 12 mm, peritumoural 15mm), the clinical model, and the six-parameter radiation-clinical omics models that incorporates clinical factors, and evaluated the performance of all the models. **Table 3** displays the AUC, accuracy, sensitivity, specificity, PPV, and NPV of various models. In the training cohort, the peritumoural 9mm model (AUC= 0.785) outperformed the clinical model (AUC= 0.772) in terms of postoperative recurrence. When clinical features were added to the peritumoural 9mm model, the combined radiation-clinical omics model’s AUC significantly improved in the training cohort (0.865), internal validation cohort (0.902), and external validation cohort (0.830) (p<0.001). **Figure 5** shows the ROC curves for the peritumoural 9mm model, the clinical model, and the combined radiation-clinical omics models in the training cohort, internal validation cohort, and external validation cohort. In order to develop a clinically applicable and more accurate model for predicting postoperative recurrence in stage ILUAD, we used the LR algorithm to construct a peritumoural 9mm radiomics nomogram incorporating some of the independent risk factors (**Figure 6**).

**Table 3:** Diagnostic effectiveness of different models.

	AUC (95%CI)	Accuracy	Sensitivity	Specificity	PPV	NPV
Training Set						
Clinical model	0.772 (0.723-0.820)	0.707	0.791	0.673	0.495	0.888
Tumor body	0.762 (0.711-0.813)	0.659	0.809	0.599	0.449	0.885
Peritumoral 3mm	0.763 (0.711-0.815)	0.632	0.852	0.542	0.430	0.901
Peritumoral 6mm	0.708 (0.651-0.766)	0.717	0.548	0.785	0.508	0.811
Peritumoral 9mm	0.785 (0.734-0.837)	0.724	0.757	0.711	0.515	0.878
Peritumoral 12mm	0.677 (0.616-0.739)	0.654	0.722	0.627	0.439	0.848
Peritumoral 15mm	0.791 (0.744-0.838)	0.714	0.687	0.725	0.503	0.851
Tumor body+Clinic	0.855 (0.817-0.893)	0.757	0.896	0.701	0.548	0.943
Peritumoral 3mm+Clinic	0.861 (0.823-0.899)	0.799	0.765	0.813	0.624	0.895
Peritumoral 6mm+Clinic	0.836 (0.794-0.878)	0.779	0.783	0.778	0.588	0.898
Peritumoral 9mm+Clinic	0.865 (0.824-0.906)	0.832	0.730	0.873	0.700	0.889
Peritumoral 12mm+Clinic	0.851 (0.810-0.892)	0.820	0.696	0.870	0.684	0.876
Peritumoral 15mm+Clinic	0.855 (0.816-0.895)	0.767	0.809	0.750	0.567	0.906
Internal Test Set						
Clinical model	0.779 (0.703-0.855)	0.737	0.760	0.727	0.535	0.880
Peritumoral 9mm	0.815 (0.742-0.888)	0.813	0.640	0.884	0.696	0.856
Peritumoral 9mm+Clinic	0.902 (0.851-0.953)	0.871	0.720	0.934	0.818	0.890
External Test Set						
Clinical model	0.773 (0.732-0.814)	0.721	0.764	0.704	0.512	0.880
Peritumoral 9mm	0.712 (0.603-0.820)	0.795	0.424	0.940	0.737	0.806
Peritumoral 9mm+ Clinic	0.830 (0.751-0.908)	0.821	0.727	0.857	0.667	0.889

**Figure 7:**
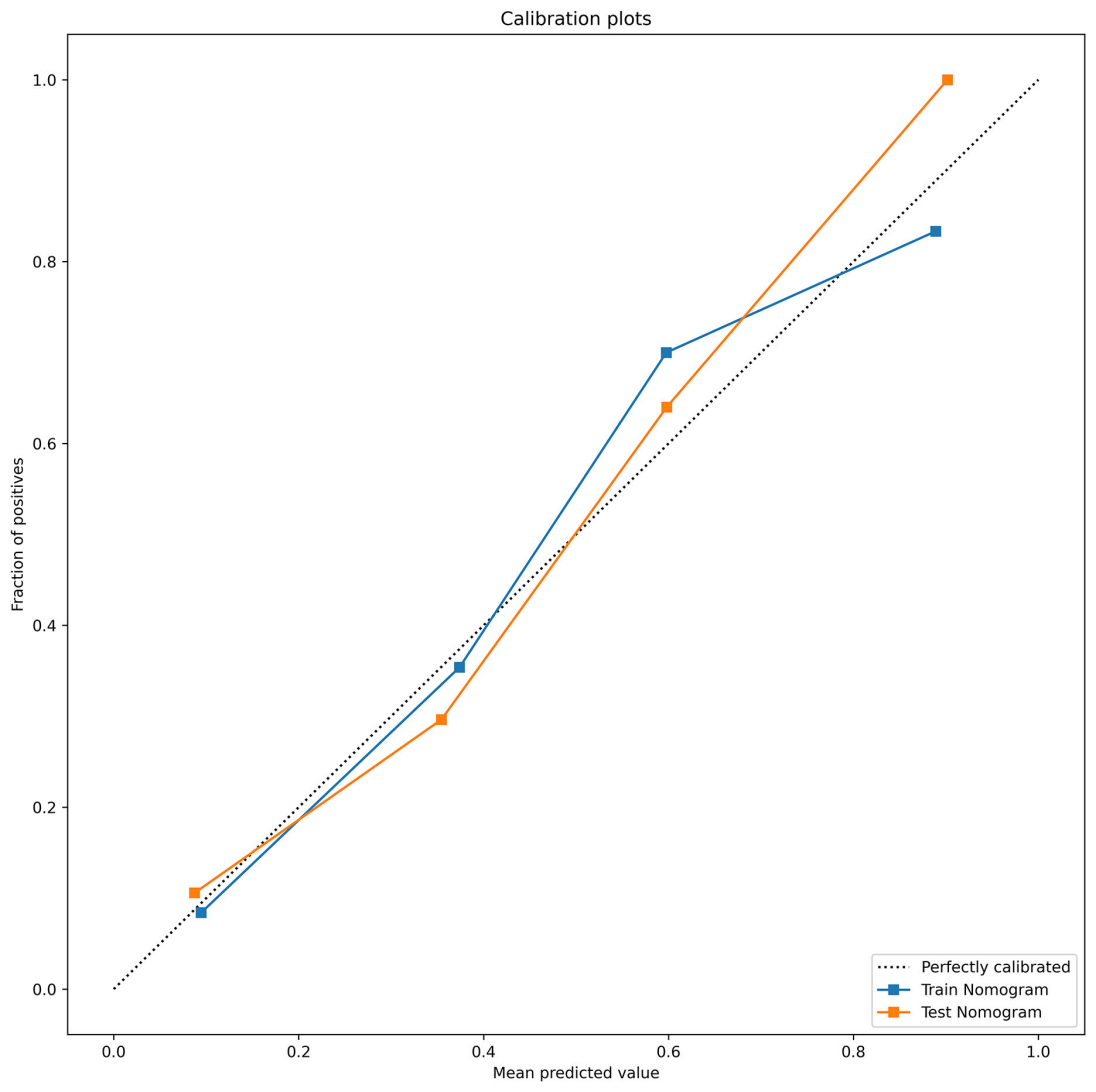
The calibration curves of combined radiation-clinical omics model for training cohort (blue dashed line) and internal validation cohort (orange dotted line).

**Figure 8:**
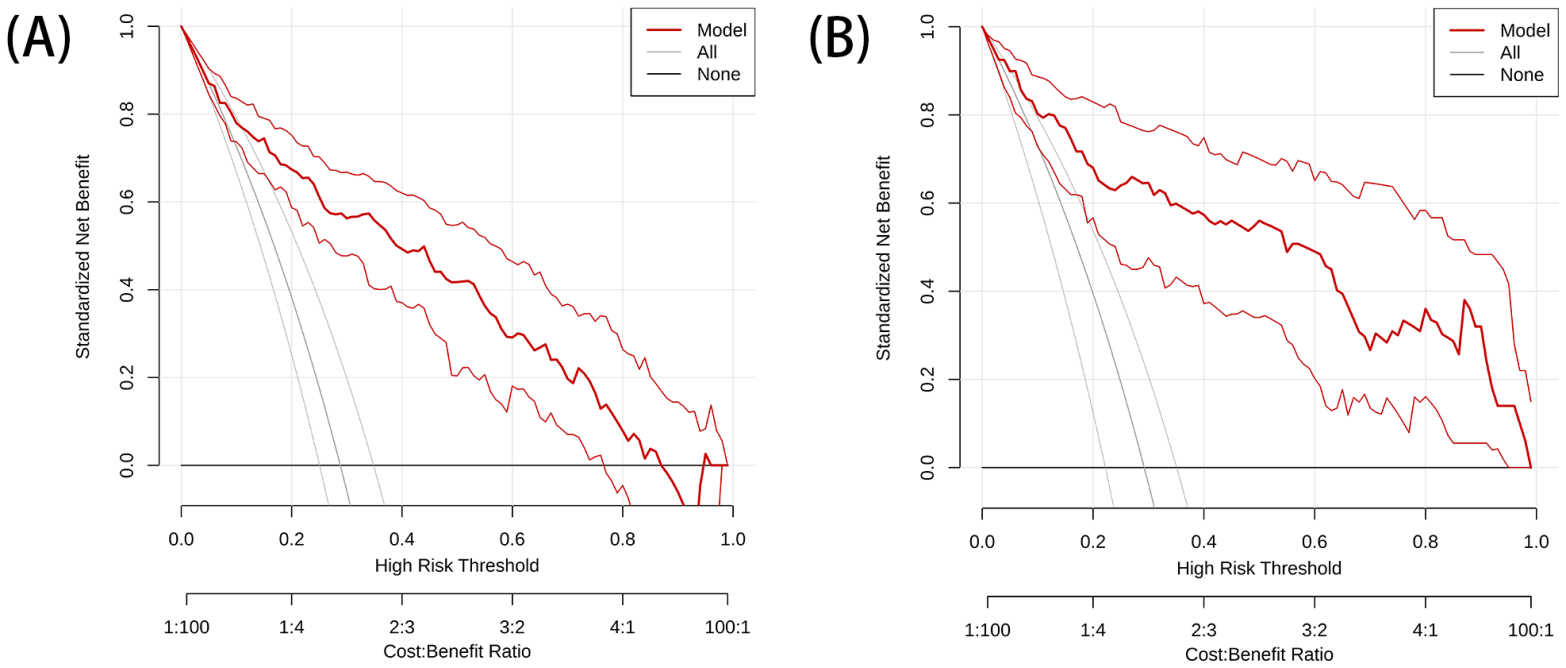
The DCA of training cohort **(A)** and internal validation cohort **(B)**.

The DeLong test showed that the AUC values of the nomogram were significantly different from those of the other models in the training cohort (P < 0.05). The combined radiation-clinical omics model’s ROC curves were significantly better than those of the radiomics and clinical models. The calibration curves of the training cohort, and the internal validation cohort in the joint model showed significant agreement in predicting postoperative recurrence in stage ILUAD (**Figure 7**). The DCA of the training cohort, and the internal validation cohort, showed that the nomogram of the combined radiation-clinical omics model had a good net clinical benefit (**Figure 8**), suggesting that it is a reliable clinical tool for predicting recurrence after surgery for stage ILUAD.

## 4 Discussion

In recent years, much attention has been paid to an emerging technology, radiomics, which automatically extracts a large number of imaging features from medical imaging data in a high-throughput manner; it appears to offer an almost unlimited range of imaging biomarkers, and shows great potential in oncology for detecting, diagnosing, evaluating prognosis, and predicting response to treatment (24–26). Furthermore, an increasing number of scholars are conducting radiomics studies on the interstitium of peripheral lung cancer, which refers to the tissue surrounding the primary tumour, and achieving favourable outcomes (17, 27, 28). This demonstrates the importance of the peritumoural region in radiomics analysis (29).

Tumour radiomics is widely used for prognostic prediction in LUAD (27). However, few studies have applied peritumoural imaging features to aid in the prediction of stage ILUAD, and the selection of the peritumoural region remains controversial. Previous studies have defined the peritumoural region as ranging from 1.5 to 20 mm (8, 30, 31). Wu et al. concluded that peritumour radiomic features based on CT images are reliable for predicting the prognosis of non-small cell carcinoma (28). The study also noted that the peritumoural region should ideally extend 15 mm, 20 mm or 30 mm from the tumour border. Chen et al. measured the bulk tumour volume as well as the bulk tumour volume in the peritumoural 3mm, peritumoural 6mm and peritumoural 9mm regions by extracting the radiomic feature regions (18), and finally constructed the bulk tumour volume of peritumoural 9mm region based on the extraction of the radiomics features had the highest AUC (training set = 0.82, internal validation = 0.75, external validation = 0.67). Liu et al. conducted another study where they extracted radiomics features from intratumoural to peritumoural 3mm, peritumoural 3mm and peritumoural 6mm regions (17). The study demonstrated that features from the intratumoural 3mm to peritumoural 3mm region had higher predictive performance. In a study using radiomics to predict early recurrence, Wang et al. selected 2.1 mm, 4.2 mm, and 8.4 mm as the peritumoural regions, extracted 2D and 3D deep learning image features, and constructed a radiomics model via an air cavity diffusion model, which resulted in good performance in both internal validation cohort and external validation cohort, demonstrating its potential for assisting in post-surgical treatment strategies (7). Wang et al. investigated 8 models of tumour perimeter 5mm, 10mm, 15mm, 20mm as well as tumour-perimeter 5mm, tumour-perimeter 10mm, tumour-perimeter 15mm, tumour-perimeter 20mm, and found that nomogram based on the combined model of tumour-perimeter 10mm and clinical features had a high predictive efficiency for STAS status in NSCLC patients (32). It can be seen that the researchers chose different peritumoural regions, but the best performing peritumoural features essentially consisted of features in the 3-9mm peritumoural regions. In addition, it was also found in previous studies that only intratumoural features were used to predict the prognosis of LUAD (33–36), whereas in this study, the use of peritumoural features performed well.

Based on these previous studies, we selected peritumoural 3mm, peritumoural 6mm, peritumoural 9mm, peritumoural 12mm and peritumoural 15mm as peritumoural regions, but unlike them, we used low-dose lung cancer screening CT plain images and performed 2.5D radiological feature extraction. In this study, we found that the combined peritumoural 9mm radiation-clinical omics model had the highest diagnostic efficacy (AUC=0.865) compared to the tumour and the rest of the peritumour models, with good AUC and sensitivity, specificity, NPV, and PPV in both the internal validation cohort and the external validation cohort, and that it outperformed the conventional unimodal model. By combining a peritumoural 9mm radiomics model with clinical factors, we have also created a visual nomogram with high predictive power and net benefit in the evaluation of recurrence after surgery for stage ILUAD. Our study provides a new approach to prognostic assessment, helps to adjust the treatment plan for patients with stage ILUAD, and enables AI-personalised management of the prognosis of these patients.

Multifactorial logistic regression analysis identified T-stage, neuron-specific enolase assay, Ki67 and nodule type as independent predictors of recurrence after surgery for stage ILUAD, which can be used for clinical modelling. Higher clinical stage, Ki67 percentage, and percentage of nodal solid component imply higher proliferation and invasiveness of tumour cells and higher risk of postoperative recurrence, which is consistent with previous reports (37–39). In addition, multifactorial logistic regression showed that neuron-specific enolase assay and nodule type were also independent predictors of postoperative recurrence, but the clinical-omics features were not significant; therefore, we developed a nomogram combining some of the independent predictors in combination with peritumoural 9mm radiomic features to predict the probability of recurrence in patients with stage ILUAD. In clinical practice, the patient’s clinical information and radiological score(radscore) are added to the nomogram to obtain multiple probability scales, and then the total score of the nomogram is calculated, which shows the probability of recurrence. Notably, there was a significant improvement in the AUC of the nomogram compared to a single radiomics and clinical model. It can gain valuable treatment time for patients with stage ILUAD that may recur, and it can help to develop a more rational and effective treatment plan. When it is known that a patient has a high probability of recurrence after surgery, some adjuvant treatments such as chemoradiotherapy or targeted drugs can be taken to reduce the chance of recurrence.

In addition, DeLong test of AUC for each model showed that in the training cohort, the AUC values for the nomogram were significantly different from those of the peritumoural 9mm radiomics model and the clinical model (P < 0.05). The results of the study showed that the combined radiation-clinical omics model performed better than the single model, and that clinical parameters also play an important role in predicting postoperative recurrence for stage ILUAD.

The different models constructed in this study not only provide intratumoural and peritumoural biological information, but also give some guidance for clinical treatment. Furthermore, by comparing the diagnostic performance of the different peritumour models, the peritumoural 9mm model had the best predictive performance overall, possibly due to the higher reproducibility of radiomics features the further away from the intratumour area. This finding may be related to the presence of homogeneous lung parenchyma in the distal peritumoural area (31). Thus, in our study, the peritumoural 9mm model showed better predictive performance than the other models. According to the recommendations of the NCCN guidelines for NSCLC 2024, 4th edition, for most patients with NSCLC, the margin requirement is to ensure that the lung parenchyma margin distance is ≥ 2 cm or ≥ the size of the tumour nodule (40), and it was found that the peritumoural region was often extended from the tumour border to 15 mm, 20 mm, or 30 mm (30, 41, 42). However, in our study, when extending to 20 mm peritumour, we found it difficult to avoid thick blood vessels and bronchioles, and complex extrapulmonary tissues, so we only extended to 15 mm peritumour.

## 5 Conclusions and limitations

This study has several limitations. Firstly, it is a retrospective study and there may be recurrent cases in the 2018 cases so far. Secondly, the sample size in this study was small and the predictive efficiency of the external validation cohort may be erroneous, and due to the small sample size, we could not perform survival analysis, and more large sample studies are needed for further validation in the future.

In summary, the combined 2.5D peritumoural 9mm radiation-clinical omics model is more accurate than the tumour and the rest of the peritumoural model in predicting the prognosis of clinical stage ILUAD, and may serve as an effective non-invasive predictive tool, which may provide value in decision-making and defining personalised treatments. However, since most of the studies were conducted retrospectively, further prospective, multicentre and biologically relevant studies based on prospective, multicentre and biologically relevant studies should be carried out in order to facilitate its clinical application.

## Data Availability

The raw data supporting the conclusions of this article will be made available by the authors, without undue reservation.

## References

[1] Siegel RL Miller KD Wagle NS Jemal A. Cancer statistics, 2023. CA: A Cancer J Clin. (2023) 73:17–48. doi: 10.3322/caac.21763 36633525

[2] Thai AA Solomon BJ Sequist LV Gainor JF Heist RS. Lung cancer. Lancet. (2021) 398:535–54. doi: 10.1016/s0140-6736(21)00312-3 34273294

[3] Siegel RL Giaquinto AN Jemal A. Cancer statistics, 2024. CA: A Cancer J Clin. (2024) 74:12–49. doi: 10.3322/caac.21820 38230766

[4] Mouabbi JA Hadid TH Uh E. Effectiveness of low-dose ct scan for lung cancer screening in the community setting. J Clin Oncol. (2019) 37:1543-. doi: 10.1200/jco.2019.37.15_suppl.1543

[5] Jacobs CD Jafari ME. Early results of lung cancer screening and radiation dose assessment by low-dose ct at a community hospital. Clin Lung Cancer. (2017) 18:e327–e31. doi: 10.1016/j.cllc.2017.01.011 28237242

[6] Chansky K Detterbeck FC Nicholson AG Rusch VW Vallières E Groome P. The iaslc lung cancer staging project: external validation of the revision of the tnm stage groupings in the eighth edition of the tnm classification of lung cancer. J Thorac Oncol. (2017) 12:1109–21. doi: 10.1016/j.jtho.2017.04.011 28461257

[7] Wang Y Ding Y Liu X Li X Jia X Li J. Preoperative ct-based radiomics combined with tumour spread through air spaces can accurately predict early recurrence of stage I lung adenocarcinoma: A multicentre retrospective cohort study. Cancer Imaging. (2023) 23:83. doi: 10.1186/s40644-023-00605-3 37679806 PMC10485937

[8] Beig N Khorrami M Alilou M Prasanna P Braman N Orooji M. Perinodular and intranodular radiomic features on lung ct images distinguish adenocarcinomas from granulomas. Radiology. (2019) 290:783–92. doi: 10.1148/radiol.2018180910 PMC6394783 30561278

[9] Gu Y She Y Xie D Dai C Ren Y Fan Z. A texture analysis-based prediction model for lymph node metastasis in stage ia lung adenocarcinoma. Ann Thorac Surg. (2018) 106:214–20. doi: 10.1016/j.athoracsur.2018.02.026 29550204

[10] Yang H Wang L Shao G Dong B Wang F Wei Y. A combined predictive model based on radiomics features and clinical factors for disease progression in early-stage non-small cell lung cancer treated with stereotactic ablative radiotherapy. Front Oncol. (2022) 12:967360. doi: 10.3389/fonc.2022.967360 35982975 PMC9380646

[11] Tan AC Tan DSW. Targeted therapies for lung cancer patients with oncogenic driver molecular alterations. J Clin Oncol. (2022) 40:611–25. doi: 10.1200/jco.21.01626 34985916

[12] Zhang X Lu B Yang X Lan D Lin S Zhou Z. Prognostic analysis and risk stratification of lung adenocarcinoma undergoing egfr-tki therapy with time-serial ct-based radiomics signature. Eur Radiol. (2022) 33:825–35. doi: 10.1007/s00330-022-09123-5 PMC9889474 36166088

[13] Gong J Bao X Wang T Liu J Peng W Shi J. A short-term follow-up ct based radiomics approach to predict response to immunotherapy in advanced non-small-cell lung cancer. OncoImmunology. (2022) 11:2028962. doi: 10.1080/2162402x.2022.2028962 35096486 PMC8794258

[14] Nardone V Boldrini L Grassi R Franceschini D Morelli I Becherini C. Radiomics in the setting of neoadjuvant radiotherapy: A new approach for tailored treatment. Cancers. (2021) 13:3590. doi: 10.3390/cancers13143590 34298803 PMC8303203

[15] Burrell RA McGranahan N Bartek J Swanton C. The causes and consequences of genetic heterogeneity in cancer evolution. Nature. (2013) 501:338–45. doi: 10.1038/nature12625 24048066

[16] Rami-Porta R Asamura H Travis WD Rusch VW. Lung cancer — Major changes in the american joint committee on cancer eighth edition cancer staging manual. CA: A Cancer J Clin. (2017) 67:138–55. doi: 10.3322/caac.21390 28140453

[17] Liu K Li K Wu T Liang M Zhong Y Yu X. Improving the accuracy of prognosis for clinical stage I solid lung adenocarcinoma by radiomics models covering tumor per se and peritumoral changes on ct. Eur Radiol. (2021) 32:1065–77. doi: 10.1007/s00330-021-08194-0 34453574

[18] Chen Q Shao J Xue T Peng H Li M Duan S. Intratumoral and peritumoral radiomics nomograms for the preoperative prediction of lymphovascular invasion and overall survival in non-small cell lung cancer. Eur Radiol. (2022) 33:947–58. doi: 10.1007/s00330-022-09109-3 36064979

[19] Kim H Lee D Cho WS Lee JC Goo JM Kim HC. Ct-based deep learning model to differentiate invasive pulmonary adenocarcinomas appearing as subsolid nodules among surgical candidates: comparison of the diagnostic performance with a size-based logistic model and radiologists. Eur Radiol. (2020) 30:3295–305. doi: 10.1007/s00330-019-06628-4 32055949

[20] Liu Z Wang S Dong D Wei J Fang C Zhou X. The applications of radiomics in precision diagnosis and treatment of oncology: opportunities and challenges. Theranostics. (2019) 9:1303–22. doi: 10.7150/thno.30309 PMC6401507 30867832

[21] Khorrami M Bera K Leo P Vaidya P Patil P Thawani R. Stable and discriminating radiomic predictor of recurrence in early stage non-small cell lung cancer: multi-site study. Lung Cancer. (2020) 142:90–7. doi: 10.1016/j.lungcan.2020.02.018 PMC7141152 32120229

[22] Potter AL Costantino CL Suliman RA Haridas CS Senthil P Kumar A. Recurrence after complete resection for non-small cell lung cancer in the national lung screening trial. Ann Thorac Surg. (2023) 116:684–92. doi: 10.1016/j.athoracsur.2023.06.004 37356517

[23] Lufan C Wenjing Z Richeng W Sai F Hao L Jing Y. Darwin: A highly flexible platform for imaging research in radiology. arXiv - CS - Comput Vision Pattern Recognition. (2020). doi: arxiv-2009.00908

[24] Aerts HJWL Velazquez ER Leijenaar RTH Parmar C Grossmann P Carvalho S. Decoding tumour phenotype by noninvasive imaging using a quantitative radiomics approach. Nat Commun. (2014) 5:4006. doi: 10.1038/ncomms5006 24892406 PMC4059926

[25] Limkin EJ Sun R Dercle L Zacharaki EI Robert C Reuzé S. Promises and challenges for the implementation of computational medical imaging (Radiomics) in oncology. Ann Oncol. (2017) 28:1191–206. doi: 10.1093/annonc/mdx034 28168275

[26] Gillies RJ Kinahan PE Hricak H. Radiomics: images are more than pictures, they are data. Radiology. (2015) 278:563–77. doi: 10.1148/radiol.2015151169 PMC4734157 26579733

[27] Choe J Lee SM Do K-H Kim S Choi S Lee J-G. Outcome prediction in resectable lung adenocarcinoma patients: value of ct radiomics. Eur Radiol. (2020) 30:4952–63. doi: 10.1007/s00330-020-06872-z 32356158

[28] Wu L Lou X Kong N Xu M Gao C. Can quantitative peritumoral ct radiomics features predict the prognosis of patients with non-small cell lung cancer? A systematic review. Eur Radiol. (2022) 33:2105–17. doi: 10.1007/s00330-022-09174-8 PMC9935659 36307554

[29] Zhuo Y Feng M Yang S Zhou L Ge D Lu S. Radiomics nomograms of tumors and peritumoral regions for the preoperative prediction of spread through air spaces in lung adenocarcinoma. Trans Oncol. (2020) 13:100820. doi: 10.1016/j.tranon.2020.100820 PMC7334418 32622312

[30] Tunali I Hall LO Napel S Cherezov D Guvenis A Gillies RJ. Stability and reproducibility of computed tomography radiomic features extracted from peritumoral regions of lung cancer lesions. Med Phys. (2019) 46:5075–85. doi: 10.1002/mp.13808 PMC6842054 31494946

[31] Lee H-S Dou TH Coroller TP van Griethuysen JJM Mak RH Aerts HJWL. Peritumoral radiomics features predict distant metastasis in locally advanced nsclc. PloS One. (2018) 13:e0206108. doi: 10.1371/journal.pone.0206108 30388114 PMC6214508

[32] Wang Y Lyu D Hu L Wu J Duan S Zhou T. Ct-based intratumoral and peritumoral radiomics nomograms for the preoperative prediction of spread through air spaces in clinical stage ia non-small cell lung cancer. J Imaging Inf Med. (2024) 37:520–35. doi: 10.1007/s10278-023-00939-1 PMC11031508 38343212

[33] Wang X Zhao X Li Q Xia W Peng Z Zhang R. Can peritumoral radiomics increase the efficiency of the prediction for lymph node metastasis in clinical stage T1 lung adenocarcinoma on ct? Eur Radiol. (2019) 29:6049–58. doi: 10.1007/s00330-019-06084-0 30887209

[34] Coroller TP Agrawal V Narayan V Hou Y Grossmann P Lee SW. Radiomic phenotype features predict pathological response in non-small cell lung cancer. Radiotherapy Oncol. (2016) 119:480–6. doi: 10.1016/j.radonc.2016.04.004 PMC4930885 27085484

[35] Traverso A Wee L Dekker A Gillies R. Repeatability and reproducibility of radiomic features: A systematic review. Int J Radiat OncologyBiologyPhysics. (2018) 102:1143–58. doi: 10.1016/j.ijrobp.2018.05.053 PMC6690209 30170872

[36] Muñoz-Barrutia A Grove O Berglund AE Schabath MB Aerts HJWL Dekker A. Quantitative computed tomographic descriptors associate tumor shape complexity and intratumor heterogeneity with prognosis in lung adenocarcinoma. PloS One. (2015) 10:e0248541. doi: 10.1371/journal.pone.0118261 PMC4349806 25739030

[37] Zhang L Liu J Yang D Ni Z Lu X Liu Y. A nomogram based on consolidation tumor ratio combined with solid or micropapillary patterns for postoperative recurrence in pathological stage ia lung adenocarcinoma. Diagnostics. (2023) 13:2376. doi: 10.3390/diagnostics13142376 37510119 PMC10378621

[38] Huang Y Liu Z He L Chen X Pan D Ma Z. Radiomics signature: A potential biomarker for the prediction of disease-free survival in early-stage (I or ii) non-small cell lung cancer. Radiology. (2016) 281:947–57. doi: 10.1148/radiol.2016152234 27347764

[39] Peng H Tan X Wang Y Dai L Liang G Guo J. Clinical significance of ki67 and circulating tumor cells with an epithelial-mesenchymal transition phenotype in non-small cell lung cancer. Am J Trans Res. (2020) 14:4085. doi: 10.1038/s41598-023-48307-x PMC7344100 32655819

[40] Riely GJ Wood DE Ettinger DS Aisner DL Akerley W Bauman JR. Non–small cell lung cancer, version 4.2024. J Natl Compr Cancer Network. (2024) 22:249–74. doi: 10.6004/jnccn.2204.0023 38754467

[41] Chellappan S Alwithenani A Bethune D Castonguay M Drucker A Flowerdew G. Profiling non-small cell lung cancer reveals that pd-L1 is associated with wild type egfr and vascular invasion, and immunohistochemistry quantification of pd-L1 correlates weakly with rt-qpcr. PloS One. (2021) 16:e0251080. doi: 10.1371/journal.pone.0251080 33956842 PMC8101740

[42] Li C Tian Y Shen Y Wen B He Y. Utility of volumetric metabolic parameters on preoperative fdg pet/ct for predicting tumor lymphovascular invasion in non–small cell lung cancer. Am J Roentgenology. (2021) 217:1433–43. doi: 10.2214/ajr.21.25814 33978465

